# Scanning electron microscopy as a method for sample visualization in protein X-ray crystallography

**DOI:** 10.1107/S2052252520003875

**Published:** 2020-04-10

**Authors:** Emma V. Beale, Anna J. Warren, José Trincão, James Beilsten-Edmands, Adam D. Crawshaw, Geoff Sutton, David Stuart, Gwyndaf Evans

**Affiliations:** a Life Science, Diamond Light Source, Harwell Science and Innovation Campus, Didcot, Oxfordshire OX11 0DE, UK; b Division of Structural Biology, University of Oxford, Wellcome Centre for Human Genetics, Oxford, Oxfordshire OX3 7BN, UK

**Keywords:** microfocus X-ray diffraction, VMXm beamline, macromolecular crystallography, cryoEM, structural biology, radiation damage, scanning electron microscopy, visualization tools

## Abstract

Developing methods to determine high-resolution structures from micrometre- or even submicrometre-sized protein crystals has become increasingly important in recent years. This applies to both large protein complexes and membrane proteins, where protein production and the subsequent growth of large homogeneous crystals is often challenging, and to samples which yield only micro- or nanocrystals such as amyloid or viral polyhedrin proteins. The versatile macromolecular crystallography microfocus (VMXm) beamline at Diamond Light Source specializes in X-ray diffraction measurements from micro- and nanocrystals. Because of the possibility of measuring data from crystalline samples that approach the resolution limit of visible-light microscopy, the beamline design includes a scanning electron microscope (SEM) to visualize, locate and accurately centre crystals for X-ray diffraction experiments. To ensure that scanning electron microscopy is an appropriate method for sample visualization, tests were carried out to assess the effect of SEM radiation on diffraction quality. Cytoplasmic polyhedrosis virus polyhedrin protein crystals cryocooled on electron-microscopy grids were exposed to SEM radiation before X-ray diffraction data were collected. After processing the data with *DIALS*, no statistically significant difference in data quality was found between datasets collected from crystals exposed and not exposed to SEM radiation. This study supports the use of an SEM as a tool for the visualization of protein crystals and as an integrated visualization tool on the VMXm beamline.

## Abstract 2

Protein crystals exposed to a scanning electron microscope beam were analysed using X-ray diffraction to assess the impact of electrons on crystal quality. These experiments support the use of a scanning electron microscope as an imaging tool for locating and centring microcrystals with a view to implement this technology on the VMXm beamline at Diamond Light Source.

## 1. Introduction   

In the last decade, microfocus X-ray beamlines have facilitated advances in structural biology by providing increasingly small intense beams of X-rays. Crystal sizes on the order of tens of micrometres down to a few micrometres are now generally considered accessible, albeit challenging, targets for protein structural biology projects. Serial femtosecond crystallography X-ray free-electron laser (XFEL) approaches have also pushed this limit, using tens of thousands of microcrystals [for a review see Martin-Garcia *et al.* (2016 ▸)] and even nanocrystals (Gati *et al.*, 2017 ▸) to determine high-resolution protein structures. Still, XFEL-based techniques have their challenges including the large number of crystals required, the inability to collect rotation data, and also the expense and limited availability of XFEL beam time. Synchrotron serial crystallography methods are also developing, but again often require reasonably large numbers of crystals (Ebrahim *et al.*, 2019 ▸; Diederichs & Wang, 2017 ▸). Electron diffraction is another growing technique for structure determination from protein crystals that are a few hundred nanometres in size (Shi *et al.*, 2013 ▸; Nannenga *et al.*, 2014 ▸; Yonekura *et al.*, 2015 ▸; Clabbers *et al.*, 2017 ▸; Xu *et al.*, 2018 ▸), with an upper limit to sample thickness of ∼400–500 nm (Shi *et al.*, 2013 ▸). Focused ion-beam milling promises a way to circumvent this thickness limit by selectively obliterating excess crystal sample to give a thin (∼200 nm) lamella from which data can be collected (Duyvesteyn *et al.*, 2018 ▸; Martynowycz *et al.*, 2019 ▸). Still, cryoEM microscopes equipped with dedicated detectors and software for low-dose protein electron-diffraction studies are reasonably scarce.

The versatile macromolecular crystallography microfocus (VMXm) beamline at Diamond Light Source, part of the VMX beamline suite, aims to further increase the scope of crystal sizes available to synchrotron-based X-ray crystallography. VMXm is designed to enable the collection of rotation datasets from crystals measuring down to 0.5 µm in size, thereby reducing the sample material required for protein structure determination, compared with serial methods, by improving the quality of data recorded from each individual crystal. In addition, crystals measuring several micrometres or less may encounter a reduced rate of radiation damage during X-ray diffraction experiments by harnessing potential photoelectron escape effects (Nave & Hill, 2005 ▸). A discussion from Holton & Frankel (2010 ▸) suggested that it is possible, under ideal conditions, to determine a 2.0 Å resolution structure from a single spherical crystal of lysozyme protein with a diameter of ∼1.2 µm. This simulation ignored all contributions to background scatter arising from dis­ordered solvent within the crystal. VMXm aims to close the gap between theory and what is currently possible in macromolecular crystallography using synchrotron X-rays. To date and to our knowledge, the smallest crystals measured using the rotation method at a synchrotron to yield a structure were reported by Ginn *et al.* (2015 ▸), where diffraction data from 768 ∼1.0 µm^3^ sized crystals were recorded at Diamond beamline I24, analysed and merged to produce a dataset complete to 2.2 Å resolution.

The VMXm beamline optics will deliver a focused variable vertical X-ray beam size of 0.3–10 µm using a single custom-profiled fixed focal-length mirror (Laundy *et al.*, 2016 ▸). Horizontal beam sizes of 0.5–5 µm are to be achieved using a two-stage demagnification scheme and a variable secondary source aperture. The horizontally deflecting double-crystal monochromator permits energies between 6 and 28 keV and, depending on the optical configuration, will deliver between 10^11^ and 10^12^ photons s^−1^ to the sample when operating at 12 keV. Samples for VMXm will typically be prepared on electron-microscopy grids using techniques borrowed from cryoEM. To further improve the signal to noise of the diffracted X-rays, the sample environment will be held under a vacuum of ∼10^−6^ mbar. As of January 2020, the major construction of the beamline has been completed, with commissioning of its components ongoing.

Collecting rotation data, as opposed to single still images, from protein crystals measuring less than a micrometre poses many practical challenges beyond the obvious radiation-damage limitations, in particular, locating and centring crystals of this size to the X-ray beam. To enable rotation data collection from crystals in this size range, VMXm aims to produce both a beam position and a sample position, stable to within 50 nm. These design specifications impose high accuracy and resolution imaging of the sample position to ensure coincidence of the beam and sample. Therefore, to align and visualize micro- and nanocrystals, which could be below the resolving power of an optical-light microscope, a scanning electron microscope (SEM) has been incorporated into the VMXm endstation sample environment. Although other methods for visualizing and centring protein crystals have been explored elsewhere (for a review see Becker *et al.*, 2017 ▸), the superior resolving quality of an SEM and the independence of SEM image quality from crystal space group, morphology, orientation and protein sequence, formed the basis of this design decision. One consideration in using an SEM in this way, however, is the potential for damage to the samples resulting from electron interactions. In an analysis by Hattne *et al.* (2018 ▸), the global and site-specific radiation damage resulting from the use of a 200 keV electron beam suggested that an incident electron dose of ∼3 e^−^ Å^−2^ resulted in the loss of high-resolution information (classed as reflections of 3 Å resolution and above). This is in line with previous analyses which have assessed electron-induced radiation damage of protein crystals (Chiu, 2006 ▸; Henderson, 1995 ▸).

CryoSEM applications for uncoated biological samples use excitation energies with orders of magnitude lower than those in the transmission electron microscopy (TEM) methods described by Hattne *et al.* (2018 ▸). Instead of needing to penetrate through the entire crystal volume as in TEM-based experiments, the SEM beam needs only to interact with the surface layer of the crystal for image formation. Although there is little published data for SEM interaction volumes of protein crystals when using low (<5 keV) incident energies of electrons, a Kanaya–Okayama estimation of the interaction hemisphere of pure amorphous carbon is ∼110 nm at 2 keV (Kanaya & Okayama, 1972 ▸). Monte Carlo simulations carried out by Barnett *et al.* suggest that the penetration depth of 2 keV electrons in water ice is ∼150 nm, although further experiments by the same group suggest these simulations perhaps underestimate this depth (Barnett *et al.*, 2012 ▸). Finally, simulations of the interaction of 2 keV electrons with graphene-coated chitin provided a maximum penetration depth of 140 nm (Park *et al.*, 2016 ▸). Given these data, the interaction depth of a 2 keV electron within a protein crystal is likely to be of the order of 100 to 200 nm.

In this study, polyhedra protein crystals from *Lymantria dispar* cytoplasmic polyhedrosis virus (CPV14) were imaged using an offline SEM, the column of which is to be integrated directly into the VMXm endstation to enable future visualization and centring of protein crystals. X-ray diffraction data were collected subsequently from these same SEM-imaged crystals. The aim was to identify whether collecting SEM images was detrimental to the diffraction quality of CPV14 crystals. This was carried out by assessing whether any significant difference was observable between diffraction data measured from crystal samples exposed to electrons versus those that were not. We demonstrate that low-dose SEM imaging is a viable method for accurately locating and aligning protein crystals without impacting the diffraction quality prior to X-ray data collection.

## 2. Materials and methods   

### 2.1. Monte Carlo simulations   

The program *CASINO* (Hovington *et al.*, 1997 ▸; Drouin *et al.*, 2007 ▸) was used to simulate the trajectory and penetration depth of 2 keV electrons in a protein crystal. A total of 200 electrons were simulated as a 10 nm beam. The protein crystal sample was described as 1000 nm thick with the formula C_1284_H_2695_N_351_O_748_S_12_ and a density of 1.35 g cm^−3^. This stoichiometry emulates the chemical composition of crystals of CPV14 with 22% solvent content [PDB ID 5a96 (Ji *et al.*, 2015 ▸)].

### 2.2. Protein preparation and crystallization   

CPV14 polyhedra were expressed and purified as described previously (Hill *et al.*, 1999 ▸; Anduleit *et al.*, 2005 ▸; Ji *et al.*, 2015 ▸). Purified cubic CPV14 crystals measured 2–4 µm in each dimension and were stored as a slurry in H_2_O at 4°C.

### 2.3. Sample mounting   

The CPV14 crystal slurry was diluted 1 in 12 into a solution of ethyl­ene glycol to give a final ethyl­ene glycol concentration of 50%(*v*/*v*). Ethyl­ene glycol was added to allow for finer control over the subsequent blotting process and to ensure cryoprotection of the crystals.

Crystals were cryocooled on electron-microscopy grids in preparation for further analysis. Cu 200 mesh grids coated with Quantifoil R 2/2 carbon film (Quantifoil) or Cu 400 mesh H7 finder grids with holey carbon (AgarScientific) were glow discharged before application of the sample. A 2 µl aliquot of 50%(*v*/*v*) ethyl­ene glycol was applied to the Cu side of the grid, followed by application of 2 µl of the diluted crystal slurry onto the carbon film. The grid was then blotted for 3.0–5.5 s from the Cu side of the grid using a Leica EM GP (20°C, humidity 90%). Blotted grids were then plunge frozen in liquid ethane. Grids were stored under liquid nitro­gen until required.

### 2.4. Sample treatment   

The samples were divided into four treatment groups: untreated, SEM loaded, SEM unexposed and SEM exposed, the details of which are described in Sections 2.4.1–2.4.3. Tests to assess radiation damage as a result of electron-beam exposure were performed using a JEOL JSM-IT100 SEM equipped with a Quorum PP3000T cryostage and cryotransfer system. The PP3000T cryostage, preparation stage (prepstage) and anticontaminator were cooled to −180°C, −180°C and −190°C, respectively. A gold-coated copper Zeiss scanning TEM shuttle was used to hold the samples during these experiments.

#### 2.4.1. Untreated   

Untreated samples were plunge frozen in liquid ethane and stored in liquid nitro­gen as detailed in Section 2.3.

#### 2.4.2. SEM loaded   

SEM-loaded samples were additionally transferred into the SEM using the cryotransfer system. Plunge-frozen samples were loaded into the shuttle under liquid nitro­gen. The cryotransfer system was used to transfer the samples into the cooled preparation chamber of the SEM. The shuttle was placed on the prepstage for 30 s before transfer onto the SEM stage for 2 mins. The shuttle was then retracted back onto the prepstage for a further 30 s before transfer out of vacuum into liquid nitro­gen using the cryotransfer system. The sample was then removed from the shuttle and stored under liquid nitro­gen.

#### 2.4.3. SEM unexposed and SEM exposed   

Crystals for the SEM-exposed and SEM-unexposed X-ray diffraction experiments were all on the same grid to control for inter-grid sample variation because of grid handling. These grids were treated in the same manner as SEM-loaded samples (see Section 2.4.2); however, instead of the 2 min incubation on the SEM stage, the grids were kept on this stage for ∼1.5 h whilst SEM exposures were carried out. SEM-exposed crystals were imaged at an accelerating voltage of 2 kV, a probe current of 40 (arbitrary units) and a working distance of 10 mm. To help with navigation around the grid and to assess grid quality, a global image of the grid was taken at 30× magnification using a 0.5 s acquisition time (total dose, 4.6 × 10^−8^ e^−^ Å^−2^). A single grid square was then used to optimize focus and astigmatism. The optimum parameters were those which provided the sharpest image as judged by eye. Image contrast and brightness were optimized using the autocontrast and brightness feature of the *InTouchScope* software package (JEOL). Images of individual grid squares containing crystals were taken at 1900× magnification using a 20 s acquisition time (7.6 × 10^−3^ e^−^ Å^−2^). Between 50 and 75 grid squares were imaged with these conditions, crystals in these images formed the SEM-exposed population. The remainder of the grid was left unexposed to electrons. Crystals in these areas formed the SEM-unexposed population. A description of the electron-dose calculations for these images can be found in the Supporting information.

### 2.5. X-ray data collection   

Electron-microscopy grids were mounted onto the beamline goniometer using a custom-made sample pin. The pin constituted a blood-vessel clip (product 14120, World Precision Instruments) on a standard magnetic pin base held in place with 3M Scotch-Weld Ep­oxy Adhesive 1838 [see Figs. S1(*a*)–S1(*c*) in the Supporting information]. Grids were transferred into the pin under liquid nitro­gen and then capped [Figs. S1(*d*)–S1(*f*)]. The capped pin was mounted onto the goniometer by hand and the cap was rapidly removed such that the grid was quickly exposed to the cryostream before liquid nitro­gen had drained from the cap.

Data were measured at Diamond Light Source beamlines I24 and I04. In all instances, data were collected as 5° wedges of contiguous data with an oscillation width of 0.1° and an exposure time of 0.05 s. Data from I24 were collected on a Dectris PILATUS3 6M detector using an X-ray beam size of 6 × 9 µm [full width at half-maximum (FWHM)] at 100% transmission and a wavelength of 0.9686 Å, producing a flux of 3.0 × 10^12^ photons s^−1^. Data from I04 were recorded using a Dectris PILATUS 6M-F detector with a beam size of 11 × 5 µm (FWHM) at 100% transmission and a wavelength of 0.9795 Å, producing a flux of 2.8 × 10^11^ photons s^−1^. For each of the four conditions, data were collected from at least three independently prepared grids. At least 100 crystals were analysed for each condition on each grid. For the SEM-exposed crystals, the electron-microscopy images were used in combination with the optical microscope views of the X-ray beamline sample position to identify the crystals that had been exposed to electrons.

### 2.6. Data processing and analysis   

In order to assess potential differences in diffraction quality, data were processed using *DIALS* (Winter *et al.*, 2018 ▸) and then analysed using *BLEND* (Foadi *et al.*, 2013 ▸). The synthesis mode of *BLEND* was then used to scale and merge the data collected from each treatment from a single grid.

In order to look for differences in initial diffraction quality between SEM-exposed and SEM-unexposed treatments, all datasets collected from the same beamline that were successfully integrated using *DIALS* were scaled together using *dials.scale*. The program *dials.cosym* was used to ensure consistent indexing prior to scaling (Gildea & Winter, 2018 ▸). The scale factor and relative *B* factor for the first image of each dataset were then extracted using *dials.python* to execute a *Python* script developed in-house.

Three replicate grids produced three complete scaled-and-merged datasets each for all four treatment groups. The mean values of key crystallographic statistics across these three replicates were compared using a one-way analysis of variance (ANOVA) method. The mean values of key statistics for the SEM-exposed and SEM-unexposed treatments were additionally compared with each other using Student’s t-tests. The distributions of scale factors and relative *B* factors for the initial images from each dataset for each of the treatment groups were compared using Kolmogorov–Smirnov (KS) tests. These statistical analyses were carried out using *GraphPad* *Prism* 8.0 (GraphPad Software, La Jolla, California, USA).

## 3. Results and discussion   

### 3.1. Monte Carlo simulations   

The average penetration depth of 2 keV electrons in a simulated CPV14 crystal was 70.0 ± 19.8 nm and the maximum penetration depth was 109.8 nm (Fig. S2). However, it should be noted that experiments by Barnett *et al.* (2012 ▸) – which assessed electron penetration depth within amorphous water-ice crystals – suggest that *CASINO* simulations may underestimate the penetration depth of electrons at these low accelerating voltages. Still, these simulations provide an estimate of the electron interaction volume for CPV14 protein crystals. On this basis, for a 2 µm CPV14 crystal (8 µm^3^), 2 keV electrons scanned across the entire surface of the crystal have the potential to penetrate, on average, ∼3.5% of the total diffracting volume. For a 0.5 µm (0.125 µm^3^) crystal, this increases to ∼14% of the total diffracting volume. This analysis does not, however, inform about the impact of electrons on diffraction quality.

### 3.2. Sample preparation and SEM exposures   

Plunge freezing the CPV14 crystals in liquid ethane using a Leica EM GP provided a reproducible method with which to mount crystals on cryoEM grids. The cuboid morphology of the crystals resulted in a preferential orientation of the crystals on the grids. The crystals generally lay with their faces parallel to the carbon film on the grids, rarely did the crystals sit on an edge or vertex. Although not explored here, methods designed by Wennmacher *et al.* (2019 ▸) have been shown to successfully combat preferential orientation of crystals on electron-microscopy grids. These methods are likely to be of particular use in future cases involving crystals from low-symmetry space groups which exhibit preferential orientation. Significant manual handling was required to transfer the plunge-frozen grids in and out of the SEM and subsequently onto the X-ray beamline whilst maintaining the samples at cryotemperatures. The combination of mechanical handling and transfer of sample grids in and out of a 1 × 10^−6^ mbar vacuum may have induced variation in sample treatments and could account for differences in crystal properties other than those caused by electron-beam exposure. In order to control for this grid-to-grid variation in crystal characteristics – which could potentially mask the effects of exposure to the electron beam – the data for SEM-exposed and SEM-unexposed crystals were taken from a single grid. For these samples, part of the grid was exposed to electrons, with the crystals in this section making up the population of SEM-exposed crystals. The remainder of the grid was not exposed to electrons and crystals in this section made up the SEM-unexposed population.

### 3.3. Data collection   

**Figure 1:**
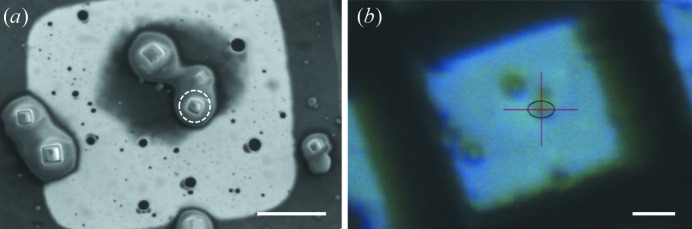
CPV14 crystals imaged using electrons and visible-light microscopy. (*a*) An example cryoSEM image of CPV14 crystals taken at an accelerating voltage of 2 kV with a working distance of 10 mm and an electron dose of 7.6 × 10^−3^ e^−^ Å^−2^. The crystals in this image formed part of the SEM-exposed treatment group. The maximum achievable resolution under these conditions with this microscope is ∼8 nm. (*b*) An image taken using the optical microscope OAV of the I24 beamline shows the corresponding grid square to that shown in panel (*a*). The maximum achievable resolution with this optical microscope is 0.7 µm. In panel (*b*), the red crosshair indicates the microfocus beam position on I24 prior to X-ray diffraction data collection from a single CPV14 crystal. The equivalent position in panel (*a*) is indicated by a dashed white circle. In both panels, the scale bar indicates 10 µm.

An example SEM image of the CPV14 crystals is shown in Fig. 1 ▸(*a*). The crystals in this image form part of the population of crystals which were exposed to electrons prior to X-ray data collection. In order to collect X-ray diffraction data from these SEM-exposed crystals, each crystal had to be located and identified on the X-ray beamline using the optical microscope on-axis-viewing system (OAV). This was achieved using ‘finder’ electron-microscopy grids (see Section 2.3) such that each individual grid square was easily identifiable and indexable under both the SEM and OAV magnification schemes. Fig. 1 ▸(*b*) depicts the corresponding OAV image for the crystals shown in the SEM image. The improvement in resolution when using an SEM is evident. It is also easier to identify the vitreous crystallization solution surrounding the individual crystals and the areas of vitreous crystallization solution close to the Cu grid bars.

To overcome the preferential orientation of the crystals on the grids, a concerted effort was made to collect data using different starting angles with respect to the orientation of the grid for the 5° wedges. The grids limited the rotation angles from which data could be collected. With the grid perpendicular to the beam, ∼±60° of data could be collected from both the ‘front’ and ‘back’ of the grid giving a total accessible range of ∼240°. Despite this limitation it was still feasible to obtain complete data because of the high symmetry of CPV14 crystals (space group *I*23).

### 3.4. Data processing and analysis   

*DIALS* was used to process the 5° wedges of data. Where data could be successfully integrated, the resultant .mtz files were fed into *BLEND*. All clusters from the analysis mode of *BLEND* were scaled and merged before a single dataset with optimal completeness was taken forward from crystals measured from each grid for further analysis. For each dataset, the high-resolution cut off was chosen based on CC_1/2_ > 0.3 (Karplus & Diederichs, 2015 ▸), which sometimes required an additional run of the program *AIMLESS* within the *BLEND* pipeline. The results of this data-processing step are presented in Tables 1 ▸ and 2 ▸.

**Figure 2:**
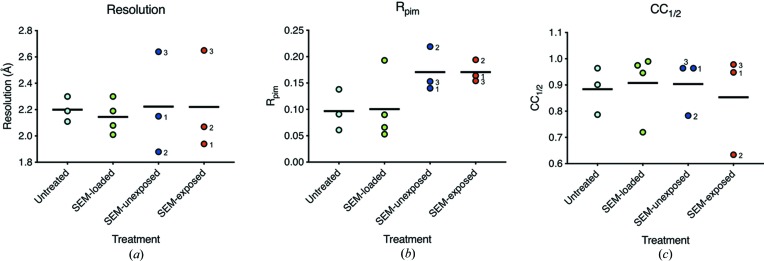
Plots of key data-processing statistics for merged datasets from the four treatment groups: untreated (cyan), SEM loaded (green), SEM unexposed (blue) and SEM exposed (red). Plots of (*a*) maximum resolution, (*b*) *R*_p.i.m._ and (*c*) CC_1/2_ show each dataset as a coloured circle and the black line indicates the mean value. For the SEM-unexposed and SEM-exposed samples, the numbers next to the circles indicate which of the three grids the data were collected from. The data from grids 1 and 2 were collected on I24, and the data from grid 3 were collected on I04.

The overall values for maximum resolution, *R*_p.i.m._ and CC_1/2_ were plotted for data collected for all four treatment groups (Fig. 2 ▸). At least three complete datasets were collected for each of the treatment groups. In the case of the SEM-exposed and SEM-unexposed datasets, complete datasets were collected for both treatment groups from each of the three replicate grids, *i.e.* one SEM-exposed and one SEM-unexposed dataset per grid, providing a total of six datasets. The mean value for each of the above-listed statistics was then calculated for the replicates of each sample treatment. The mean values for each of these statistics were compared across all treatment groups through use of a one-way ANOVA method. These analyses showed no statistically significant difference between the mean values of maximum resolution, *R*_p.i.m._ or CC_1/2_ across any of the treatment groups. A further Student’s t-test was used to compare the mean values of these statistics between the SEM-exposed and SEM-unexposed datasets. Using this method of analysis, there was no statistically significant difference (*p* > 0.05) measured between these crystallographic statistics for data collected from crystals pre-exposed to 2 keV SEM beam (SEM exposed) versus crystals that were not exposed (SEM unexposed).

**Figure 3:**
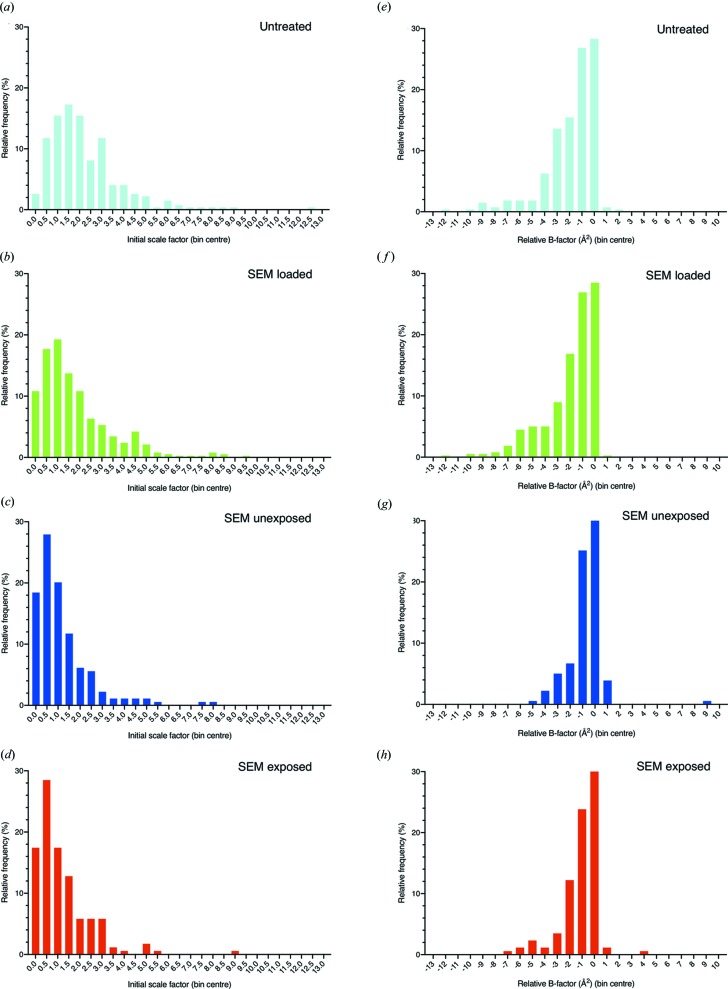
Histograms showing the initial scale factors and relative *B* factors for datasets collected from crystals across different treatments. Scale factors (*a*)–(*d*) and relative *B* factors (*e*)–(*h*) for the first frame of each dataset collected from individual CPV14 crystals were extracted following a single scaling job of all 1151 datasets with *DIALS*. These factors were then plotted as histograms, where each histogram contains the distribution of either initial scale factor or *B* factor within a given treatment group. The treatment groups were: untreated [cyan, (*a*) and (*e*)], SEM loaded [green, (*b*) and (*f*)], SEM unexposed [blue, (*c*) and (*g*)] and SEM exposed [red, (*d*) and (*h*)].

To further investigate the potential damage to the crystals caused by pre-exposure to SEM radiation the 1151 integrated datasets collected on I24 were all scaled together. This was achieved using *dials.cosym* (Gildea & Winter, 2018 ▸), to ensure a consistent indexing scheme, followed by *dials.scale*. In an attempt to assess whether the sample treatments significantly altered the initial diffraction of the crystals, both the scale factor and the relative *B* factor for the initial diffraction pattern from each dataset were extracted from the data, these values can be seen plotted as histograms for each treatment group in Fig. 3 ▸.

A comparison of these distributions between treatment groups by way of a KS test revealed that the distributions of both scale and *B* factor for SEM-unexposed and SEM-exposed treatments were not significantly different to each other (scale factors of *p* > 0.05 and *D* = 0.07175, and *B* factors of *p* > 0.05 and *D* = 0.07613) (where *D* is the KS distance). This analysis infers that the pre-exposure of the crystals to the electron dose used here did not significantly alter the diffraction quality of these crystals. Further KS tests comparing the distributions of initial scale and *B* factor between the other treatment groups were also carried out. The distributions of the scale factors for untreated samples were significantly different to the distributions of both the SEM-loaded and SEM-unexposed samples (*p* < 0.0001 in both tests). These results suggest that the grid handling involved in putting the grids into and out of vacuum at cryogenic temperatures has an effect on the diffraction quality of the crystals. Furthermore, the distributions for the SEM-loaded samples were significantly different to those of the SEM-unexposed samples (*p* < 0.0001 in all tests). This suggests that the additional time spent on the SEM cryostage in the case of the SEM-unexposed samples is having an effect on the diffraction quality of the crystals. This could be related to the vacuum environment or the cooling of the samples whilst in the SEM, or a combination of the two. An analysis of the temperature of the SEM shuttle was carried out (data not shown) indicating that the shuttle is kept below devitrification temperature during transfer and whilst on the SEM stage; however, no measurements were able to be carried out to measure the temperature of the grid itself during transfer. Given that the grid relies on thermal contact with the shuttle for effective cooling, it cannot be ruled out that inefficient thermal contact and thus insufficient cooling contributed to these significant differences. This study highlights the importance of detailed characterization of cryogenic handling workflows when dealing with sensitive biological samples.

It is important to note that CPV14 is a well diffracting sample and that other crystals, such as those formed from large molecular weight membrane proteins, might be more susceptible to radiation damage. In reference to this point, research from Holton & Frankel (2010 ▸) provides some useful discussion and offers some insight into the potential relationship between CPV14 and other potentially more disordered or radiation-sensitive proteins. Their discussion compares the test protein case of lysozyme with a large (10 MDa) protein crystal with a Wilson *B* factor of 61 Å^2^. The calculations within the article suggest that this larger protein with a Wilson *B* factor three times that of the lysozyme crystal requires a volume close to two orders of magnitude larger to produce the equivalent diffraction resolution and quality. This suggests that such a crystal is approximately two orders of magnitude more sensitive to X-ray dose than the lysozyme counterpart described in the article. The soluble nature of CPV14 and its molecular weight make it more comparable with the lysozyme example of Holton & Frankel (2010 ▸) than the 10 MDa protein. It is possible, therefore, that a more disordered or radiation-sensitive protein, for instance a membrane protein, could be approximately two orders of magnitude more sensitive to radiation damage compared with CPV14. Considering this, we believe that the incident electron doses used here still place us well within the damage threshold for even the most sensitive crystals, especially since the low-energy electrons used are predicted to penetrate no more than 150 nm into the surface of the samples.

## 4. Conclusions   

The analyses described here support the use of low-voltage SEM imaging as a method to visualize and locate micrometre-sized protein crystals prior to X-ray diffraction experiments. Using 2 keV electrons at the doses described, the results presented here indicate no significant difference between the quality of X-ray diffraction data from crystals that were exposed to the SEM beam and those that were not. This is in line with the literature which states that doses of 3 e^−^ Å^−2^ are required to cause a reduction in high-resolution reflections (described as reflections < 3 Å resolution) (Chiu, 2006 ▸; Henderson, 1995 ▸; Hattne *et al.*, 2018 ▸). These experiments were carried out using electron doses that were several orders of magnitude lower than this 3 e^−^ Å^−2^ threshold and electron energies that leave the bulk of the protein crystals unpenetrated. Indeed, the lack of statistically significant or measurable radiation damage to the SEM-exposed samples supports the use of such doses and electron energies for imaging. In conclusion, low-voltage SEM imaging is an appropriate method for the visualization and subsequent alignment of samples below the resolution of optical microscopy.

## 5. Related literature   

The following reference is cited in the Supporting information for this article: Zheng *et al.* (2009). ▸

## Supplementary Material

Supporting information. DOI: 10.1107/S2052252520003875/jt5045sup1.pdf

## Figures and Tables

**Table 1:** Data-processing statistics Values for the outer shell are given in parentheses.

	Plunge frozen			SEM loaded			
	1	2	3	1	2	3	4
Diffraction source	I24			I24			
Wavelength (Å)	0.9686			0.9686			
Temperature (K)	100			100			
Detector	PILATUS3 6M			PILATUS3 6M			
Rotation range per image (°)	0.1			0.1			
Total rotation range (°)	5.0			5.0			
Exposure time per image (s)	0.05			0.05			
Space group	*I*23			*I*23			
*a, b, c* (Å)	103.1, 103.1, 103.1	103.3, 103.3, 103.3	103.4, 103.4, 103.4	103.2, 103.2, 103.2	103.3, 103.3, 103.3	103.2, 103.2, 103.2	103.2, 103.2, 103.2
α, β, γ (°)	90, 90, 90	90, 90, 90	90, 90, 90	90, 90, 90	90, 90, 90	90, 90, 90	90, 90, 90
No. of crystals (datasets) used in analysis	58	67	60	38	62	47	77
Resolution range (Å)	72.87–2.11 (2.17–2.11)	73.05–2.30 (2.38–2.30)	73.12–2.19 (2.26–2.19)	73.00–2.19 (2.25-–2.19)	73.04–2.01 (2.06–2.01)	72.94–2.30 (2.38–2.30)	72.99–2.08 (2.14–2.08)
Total no. of reflections	244527 (20366)	223816 (22111)	281703 (24668)	135610 (11929)	265547 (19839)	139273 (13476)	315385 (23504)
No. of unique reflections	10695 (884)	8333 (802)	9608 (835)	9609 (830)	12307 (909)	8196 (799)	11121 (853)
Completeness (%)	99.7 (100.0)	99.6 (99.7)	99.7 (99.9)	99.6 (99.6)	99.6 (100.0)	98.9 (99.7)	99.7 (99.8)
Redundancy	22.9 (23.0)	26.9 (27.6)	29.3 (29.5)	14.1 (14.4)	21.6 (21.8)	17.0 (16.9)	28.4 (27.6)
CC_1/2_	0.964 (0.924)	0.901 (0.838)	0.787 (0.631)	0.990 (0.965)	0.946 (0.936)	0.720 (0.540)	0.975 (0.931)
〈*I*/σ(*I*)〉	10.6 (7.6)	9.7 (7.7)	6.7 (4.7)	12.3 (8.5)	10.2 (7.0)	5.7 (4.1)	10.1 (7.1)
*R*_p.i.m._	0.091 (0.250)	0.061 (0.092)	0.138 (0.229)	0.053 (0.098)	0.066 (0.133)	0.193 (0.393)	0.90 0.258)

**Table 2:** Data-processing statistics Values for the outer shell are given in parentheses.

	SEM 1		SEM 2		SEM 3	
	Unexposed	Exposed	Unexposed	Exposed	Unexposed	Exposed
Diffraction source	I24	I24	I24	I24	I04	I04
Wavelength (Å)	0.9686		0.9686		0.9795	
Temperature (K)	100		100		100	
Detector	PILATUS3 6M		PILATUS3 6M		PILATUS 6M-F	
Rotation range per image (°)	0.1		0.1		0.1	
Total rotation range (°)	5		5		5	
Exposure time per image (s)	0.05		0.05		0.05	
Space group	*I*23		*I*23		*I*23	
*a, b, c* (Å)	103.2, 103.2, 103.2	103.1, 103.1, 103.1	103.3, 103.3, 103.3	103.2, 103.2, 103.2	103.2, 103.2, 103.2	103.2, 103.2, 103.2
α, β, γ (°)	90, 90, 90	90, 90, 90	90, 90, 90	90, 90, 90	90, 90, 90	90, 90, 90
No. of crystals (datasets) used in analysis	56	48	36	29	41	41
Resolution range (Å)	51.58–2.15 (2.22–2.15)	72.91–1.94 (1.99–1.94)	73.04–1.88 (1.93–1.88)	72.98–2.07 (2.13–2.07)	72.96–2.64 (2.77–2.64)	72.94–2.65 (2.78–2.65)
Total no. of reflections	223880 (19857)	268471 (18290)	200883 (13291)	133011 (8973)	105759 (14453)	102334 (13738)
No. of unique reflections	10095 (878)	13649 (903)	14968 (963)	11136 (859)	5502 (724)	5425 (703)
Completeness (%)	100.0 (100.0)	100.0 (100.0)	99.8 (99.6)	98.8 (97.5)	99.5 (100.0)	99.8 (99.8)
Redundancy	22.2 (22.6)	19.7 (20.3)	13.4 (13.8)	11.9 (10.4)	19.2 (20.0)	18.9 (19.5)
CC_1/2_	0.964 (0.906)	0.948 (0.572)	0.783 (0.380)	0.634 (0.577)	0.964 (0.952)	0.978 (0.966)
〈*I*/σ(*I*)〉	6.6 (4.3)	4.2 (2.6)	3.1 (1.8)	3.2 (2.5)	10.6 (8.0)	8.8 (6.6)
*R*_p.i.m._	0.140 (0.278)	0.164 (0.372)	0.219 (0.499)	0.194 (0.190)	0.153 (0.215)	0.114 (0.211)
