# Supplementary material for: Scanning electron microscopy as a method for sample visualization in protein X-ray crystallography
Source: IUCrJ. 2020 Apr 10;7(Pt 3):500–8. doi: 10.1107/S2052252520003875 (PMC7201292; doi:10.1107/S2052252520003875)
Supplement: Supplementary file 1 [file m-07-00500-sup1.pdf]

IUCrJ

Volume 7 (2020)

Supporting information for article:

Scanning electron microscopy as a method for sample visualization in protein X-ray crystallography

Emma V. Beale, Anna J. Warren, José Trincão, James Beilsten-Edmands, Adam D. Crawshaw, Geoff Sutton, David Stuart and Gwyndaf Evans

S1. Calculating dose at the SEM sample position

A Deben SEM probe current meter was used to measure the current at the sample position. The resultant data can be found in Table S1. The measured currents were then used to calculate the electron dose at the sample position. The cylindrical opening in the gold-coated copper Zeiss STEM shuttle was used as a Faraday cup to measure the current. Currents were measured at a working distance of 10 mm using accelerating voltages of 0.5, 1, 1.5, 2, 2.5, 3, 4, 5, 6, 7, 8, 9, 10, 12, 14, 16, 18 and 20 kV. At each different accelerating voltage, probe currents of 0 to 90 in steps of 10 were measured with an additional measurement at a maximum probe current of 99. The measured currents were fed into the following equations from Zheng et al. (2009) (Zheng *et al.*, 2009).

$$N = \frac{I}{e}$$

Where N is the number of electrons hitting the sample per unit time (s^{-1}), I is the measured beam current in amperes and e is the elementary charge of an electron (1.6×10^{-19} C).

$$ED = \frac{N}{A} \cdot t$$

Where ED is the total electron dose (e Å^{-2}), A is the area of sample exposed to the electron beam (Å^{2}) and t is the exposure time for the sample under the electron beam (s). The exposure time was taken to be the dwell time (the time the electron beam spends at each pixel during an acquisition). This equates to 0.16 µs per pixel for a 0.5 s acquisition and 0.41 µs per pixel for a 20 s acquisition. For the JEOL IT-100, at 30 × magnification, each pixel measures 3.4 × 3.4 µm (1.2×10^{9} Å^{2}) and at 1900 × magnification, each pixel measures 0.053 × 0.053 µm (2.8×10^{5} Å^{2}).

Table S1 Measured currents in nanoamperes generated by the SEM beam at a working distance of 10 mm.

Voltage (kV)	Probe current (arbitrary units)										
	0	10	20	30	40	50	60	70	80	90	99
0.5	0.001	0.001	0.001	0.001	0.002	0.005	0.014	0.038	0.082	0.118	0.157
1	0.001	0.001	0.001	0.003	0.007	0.019	0.068	0.222	0.491	0.676	0.713
1.5	0.001	0.001	0.002	0.004	0.013	0.043	0.184	0.715	2.04	3.51	4.91
2	0.001	0.002	0.003	0.006	0.021	0.068	0.296	1.151	3.17	5.27	6.85
2.5	0.002	0.002	0.003	0.008	0.026	0.083	0.363	1.445	4.37	8.05	12.1
3	0.002	0.002	0.004	0.01	0.034	0.112	0.501	2.02	6.45	12.82	22.1
4	0.002	0.002	0.004	0.011	0.041	0.141	0.648	2.75	9.35	20.2	38.3
5	0.001	0.002	0.005	0.012	0.047	0.168	0.797	3.45	11.85	25.2	45.2
6	0.002	0.002	0.005	0.012	0.045	0.162	0.79	3.52	12.35	27.3	48.5
7	0.002	0.002	0.004	0.011	0.04	0.144	0.712	3.22	11.92	27.8	51.2
8	0.002	0.002	0.004	0.01	0.037	0.136	0.685	3.11	11.52	27.5	58.1
9	0.002	0.002	0.004	0.008	0.031	0.123	0.634	2.96	12.01	32.4	140
10	0.002	0.002	0.004	0.009	0.034	0.128	0.646	2.87	10.45	27.8	180
12	0.001	0.002	0.004	0.009	0.035	0.13	0.662	3.00	11.51	30.8	223
14	0.002	0.002	0.004	0.009	0.035	0.128	0.653	3.01	11.95	32.1	175
16	0.002	0.002	0.005	0.012	0.046	0.172	0.882	4.18	16.83	45.1	200
18	0.001	0.002	0.004	0.012	0.046	0.175	0.903	4.31	17.1	45	226
20	0.003	0.003	0.006	0.013	0.049	0.182	0.923	4.36	16.75	50.2	411

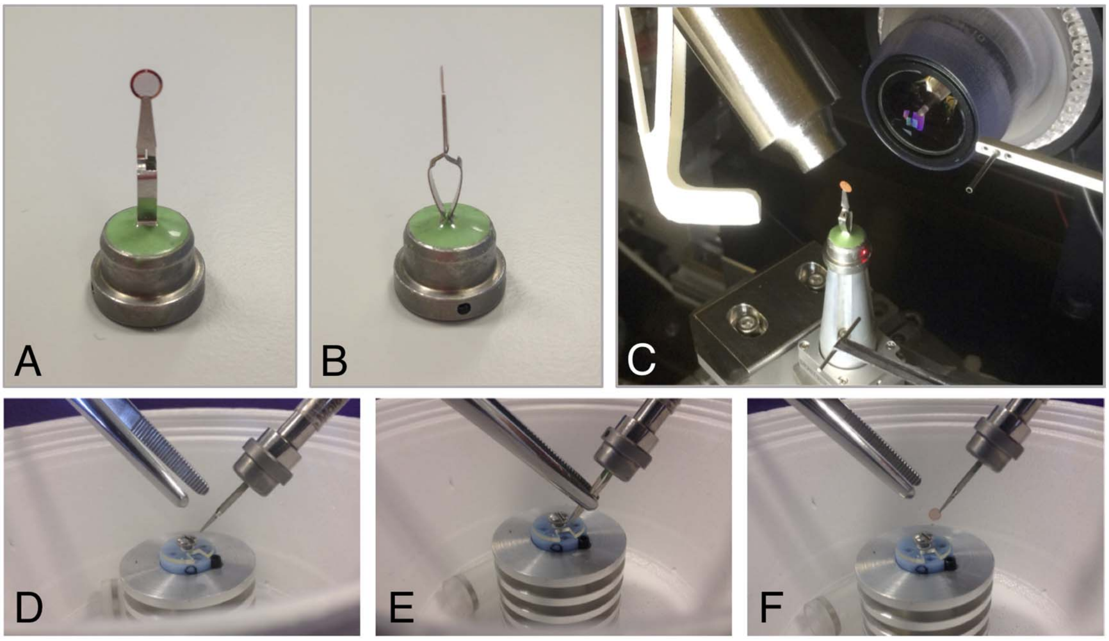

Figure S1 The custom pin mount used for loading electron microscopy grids onto the beamline goniometers. Both (A) and (B) show close up views of the pin with an electron microscopy grid mounted and (C) shows a grid mounted onto the I24 goniometer using one of these custom pin mounts. Panels (D-F) show the loading procedure used to transfer a grid from a standard storage box into the custom pin mount under liquid nitrogen. Note that liquid nitrogen has been omitted to improve the clarity of the images. (D) The grid box was held on an aluminium stand completely submerged under liquid nitrogen. (E) Large forceps were used to open the tweezers on the pin and guide it into the grid box. (F) The large forceps were released so that the grid was clamped in the pin and could be removed from the grid box whilst being kept under liquid nitrogen at all times. Once the pin was loaded with the grid, the pin could then be capped, ready for transfer to the beamline goniometer and cryostream.

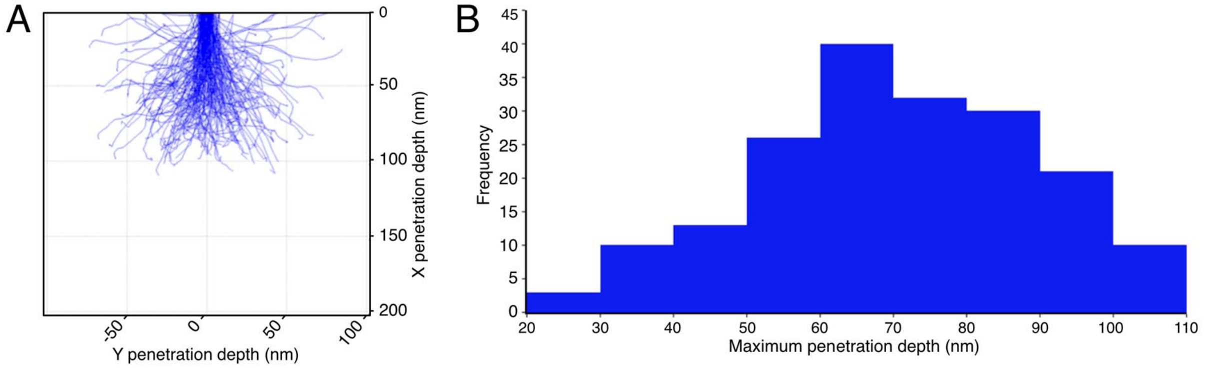

Figure S2 CASINO simulation of trajectories of 2 keV electrons through a CPV14 crystal. Panel A shows the simulated trajectories of the electrons, trajectories for backscattered electrons have been omitted. Panel B is a histogram showing the distribution of the maximum penetration depth of each electron. The largest maximum penetration depth of any electron was 109.8 nm with a mean maximum penetration depth of 70.0 ± 19.8 nm.
